# COP28 Open Letter on fossil fuels from the Global Medical and Health Community

**DOI:** 10.17305/bb.2023.9996

**Published:** 2024-02-01

Dear COP28 President-Designate Sultan Ahmed Al-Jaber,

This year, world leaders gathering in the UAE to take stock of their climate commitments will for the first time engage in official programming focused on health. We, the signatories of this letter, support your leadership in bringing health to the forefront at COP28.

As global health leaders, we are committed to achieving health and well-being for all; this is not possible without a safe and stable climate. The Paris Agreement enshrined the “right to health” as a core obligation for climate action. Yet, communities, health workers and health systems around the world already face the alarming impacts of a changing climate. Climate change-induced extreme weather events are becoming more frequent and severe; many countries are grappling with the health consequences of extreme heat, unprecedented storms, floods, food and water insecurity, wildfires and displacement. For COP28 to truly be a “health COP,” it must address the root cause of the climate crisis: the continued extraction and use of fossil fuels including coal, oil and gas. **We call on the COP28 Presidency and the leaders of all countries to commit to an accelerated, just and equitable phase-out of fossil fuels as the decisive path to health for all.**

**Ending our dangerous dependency on fossil fuels will improve the health prospects of future generations and save lives.** Keeping the global temperature increase within the 1.5 °C target of the Paris Agreement is essential to ensure good health and economic prosperity for all. This will only be possible if we rapidly phase out fossil fuels. This will limit global warming, thereby protecting health from the devastating impacts of extreme weather, and preventing further ecological degradation and biodiversity loss. Failing to do so will lead to overwhelming health consequences, as well as the loss of key natural resources and ecosystem services that are critical to both human and non-human species health, thereby undermining One Health and planetary health.

In addition to climate-related health impacts, air pollution caused in part by burning fossil fuels causes 7 million premature deaths annually. In 2019, the economic costs of air pollution-related health impacts amounted to over US$8.1 trillion, or 6.1% of global GDP. By improving air quality, governments can reduce the burden of disease from multiple cancers, heart disease, neurological conditions including stroke, and chronic and acute respiratory diseases, including asthma and chronic obstructive pulmonary disease (COPD). Investments in clean energy sources will save hundreds of billions of dollars in health care costs associated with air pollution every year, while reducing economic losses from extreme weather events with damages worth US$253 billion (in 2021).

**A full and rapid phase-out of fossil fuels is the most significant way to provide the clean air, water, and environment that are foundational to good health.** We cannot depend on inadequate solutions, like Carbon Capture and Storage (CCS), that extend the use of fossil fuels but do not generate the real and immediate health improvements which a renewable energy transition provides. False solutions like CCS risk exacerbating harmful emissions, straining the health of overburdened communities, and delaying meaningful climate action.

**The energy transition must be equitable for all.** In transitioning to a clean energy future, there is an opportunity to undo the injustices of the fossil fuel dependent system, taking a systemic approach and emphasizing health, care and community well-being, leaving no one behind. Global leaders must ensure everyone, including fragile states and the most remote and excluded communities, has access to non-polluting, affordable, reliable, accessible and resilient clean energy, as well as to emerging technologies that make best use of this energy. A just transition offers the opportunity to reduce health inequities faced by minority and marginalized communities, especially with respect to the health effects of ongoing fossil fuel use and dependence. 

**Unlocking finance is essential to deliver a healthy and just transition.** Achieving climate and health goals will only be feasible if we stop investing in fossil fuels and invest instead in proven climate and health solutions. Each year, countries spend hundreds of billions of dollars subsidizing the fossil fuel industry, money that could be spent investing in a healthy future. High-income countries, development finance institutions, and the private sector must dramatically increase, and fulfill wittheir commitments to drive investments in clean energy, clean air, and economic development for the communities most harmed by climate change and fossil fuel pollution.

**Fossil fuel interests have no place at climate negotiations.** The fossil fuel industry cannot be allowed to continue its decades-long campaign of obstructing climate action at the UNFCCC negotiations and beyond. Just as the tobacco industry is not allowed to participate in the WHO Framework Convention on Tobacco Control, it is imperative to safeguard global collaboration on climate progress from the lobbying, disinformation, and delays in favor of industry interests.

Without ambitious climate action, the burden on health care systems and workers will be insurmountable. Health gains made in recent decades will be in vain and we will see the harmful impacts of climate change ruin our chances for a safe, equitable and just future. 

In this extraordinary year, with health on the COP agenda for the first time, **we urge you to deliver real climate progress: Commit to an accelerated, just and equitable phase-out of fossil fuels and invest in a renewable energy transition as the decisive path to health for all.**

Sincerely,

*Global Health Organization Leadership (Alphabetical by organization)*

Dr. Githinji Gitahi, CEO, Amref Health Africa

Dr. Pam Cipriano, President, International Council of Nurses

Dr. Salman Khan, Liaison Officer for Public Health Issues, International Federation of Medical Students' Associations

Dr. Naveen Thacker, President, International Pediatric Association

Dr Christos Christou, International President, Médecins Sans Frontiéres

Dr. María del Carmen Calle Dávila, Executive Secretary, Organismo Andino du Salud (Andean Health Organization)

Prof. Luis Eugenio de Souza, President, World Federation for Public Health Associations

Dr. Lujain Alqodmani, President, World Medical Association

*Regional Leaders in Health (Alphabetical by surname)*

Dr. Mary T. Bassett, Director, FXB Center for Health and Human Rights, Harvard University

Dr. Fiona Godlee, Former Editor-in-chief of the British Medical Journal

Prof. (Dr.) Arvind Kumar, Chairman, Institute of Chest Surgery, Chest Onco Surgery and Lung Transplantation, Medanta Hospital, India

Dame Parveen Kumar, Emeritus Professor of Medicine and Education, Barts and The London School of Medicine and Dentistry

Dr. Lwando Maki, Secretary, Public Health Association of South Africa

Dr. Jemilah Mahmood, Executive Director, Sunway Center for Planetary Health - Malaysia

Dr. Kari C. Nadeau, MD, PhD, Chair of the Department of Environmental Health at Harvard School of Public Health

Prof. (Dr.) K Srinath Reddy, Past President of Public Health Foundation of India

This letter is supported and endorsed by:

*National Health Organization Leadership (Alphabetical by organization)*

Dr. Rosana Teresa Onocko Campos, President, Associação Brasileira de Saúde Coletiva (Brazil)

Katie Huffling, DNP, Executive Director, Alliance of Nurses for Healthy Environments (US)

Dr. Latifa Patel, Representative Body Chair, British Medical Association

Kamran Abassi, Editor-in-Chief, British Medical Journal (UK)

Dr. Frances Peart, President & Board Chair, Climate and Health Alliance (Australia)

Dr. Kate Wylie, Executive Director, Doctors for the Environment Australia

Dr. Agonafer Tekalenge, President, Ethiopian Public Health Association

Diederik Aarendonk, Forum Coordinator Global Health Organization Leadership, European Forum for Primary Care

Prof. Kevin Fenton, President, Faculty of Public Health (UK)

Dr. Ansgar Gerhardus, Board Chair, German Public Health Association

Dr. Vital Ribeiro, Chair, Associação Civil Projeto Hospitais Saudáveis (Healthy Hospitals Project)

The Board of the Public Health Association of South Africa

Diana Zeballos, Executive Secretary, Sustainable Health Equity Movement (SHEM)

Dr. Adeline Kimambo, Executive Secretary, Tanzania Public Health Association

Dr. Richard Smith, Chair, UK Health Alliance on Climate Change

